# 2,4-Diamino-6-methyl-1,3,5-triazin-1-ium tetra­fluoro­borate

**DOI:** 10.1107/S1600536811038797

**Published:** 2011-09-30

**Authors:** Sundaramoorthy Gomathi, Packianathan Thomas Muthiah

**Affiliations:** a School of Chemistry, Bharathidasan University, Tiruchirappalli 620 024, Tamilnadu, India

## Abstract

In the crystal structure of the title salt, C_4_H_8_N_5_^+^·BF_4_^−^, centrosymmetrically related cations undergo base pairing *via* a pair of N—H⋯N hydrogen bonds, forming an *R*_2_^2^(8) ring motif. The cations and anions inter­act *via* N—H⋯F hydrogen bonds, generating supra­molecular layers parallel to (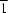
20), which are in turn linked into a three-dimensional network, forming rings of *R*_6_^6^(24) graph-set motif. The crystal structure is further stabilized by π–π stacking inter­actions [centroid–centroid distance = 3.3361 (12) Å].

## Related literature

For hydrogen-bond motifs, see: Bernstein *et al.* (1995 ▶); Etter (1990 ▶). For related structures, see: Conant *et al.* (1964 ▶); Gokul Raj *etal.* (2006 ▶); Zimmermann *et al.* (1963 ▶); Hemamalini *et al.* (2005 ▶); Balasubramani *et al.* (2007 ▶); Li *et al.* (2011 ▶). For π–π stacking inter­actions, see: Hunter (1994 ▶).
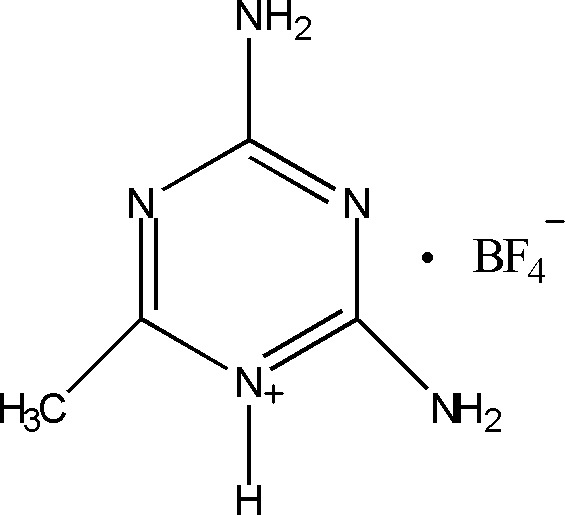


## Experimental

### 

#### Crystal data

C_4_H_8_N_5_^+^·BF_4_^−^
*M* *_r_* = 212.96
Triclinic, 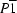

*a* = 6.9982 (3) Å
*b* = 8.2887 (4) Å
*c* = 8.5353 (4) Å
α = 63.931 (2)°
β = 83.209 (3)°
γ = 85.057 (3)°
*V* = 441.29 (4) Å^3^
*Z* = 2
Mo *K*α radiation
μ = 0.16 mm^−1^
*T* = 296 K
0.06 × 0.05 × 0.04 mm

#### Data collection

Bruker SMART APEXII CCD area-detector diffractometer
Absorption correction: multi-scan (*SADABS*; Bruker, 2008 ▶) *T*_min_ = 0.990, *T*_max_ = 0.993
8850 measured reflections
2196 independent reflections
1842 reflections with *I* > 2σ(*I*)
*R*_int_ = 0.022

#### Refinement

*R*[*F* ^2^ > 2σ(*F* ^2^)] = 0.066
*wR*(*F* ^2^) = 0.203
*S* = 1.09
2196 reflections
128 parameters
H-atom parameters constrained
Δρ_max_ = 0.52 e Å^−3^
Δρ_min_ = −0.54 e Å^−3^

### 

Data collection: *APEX2* (Bruker, 2008 ▶); cell refinement: *SAINT* (Bruker, 2008 ▶); data reduction: *SAINT*; program(s) used to solve structure: *SHELXS97* (Sheldrick, 2008 ▶); program(s) used to refine structure: *SHELXL97* (Sheldrick, 2008 ▶); molecular graphics: *PLATON* (Spek, 2009 ▶); software used to prepare material for publication: *PLATON*.

## Supplementary Material

Crystal structure: contains datablock(s) global, I. DOI: 10.1107/S1600536811038797/rz2638sup1.cif

Structure factors: contains datablock(s) I. DOI: 10.1107/S1600536811038797/rz2638Isup2.hkl

Supplementary material file. DOI: 10.1107/S1600536811038797/rz2638Isup3.cml

Additional supplementary materials:  crystallographic information; 3D view; checkCIF report

## Figures and Tables

**Table 1:** Hydrogen-bond geometry (Å, °)

*D*—H⋯*A*	*D*—H	H⋯*A*	*D*⋯*A*	*D*—H⋯*A*
N1—H1⋯F1	0.86	1.90	2.758 (2)	173
N2—H2*A*⋯F2^i^	0.86	2.01	2.800 (4)	152
N2—H2*B*⋯F4^ii^	0.86	2.02	2.877 (4)	177
N4—H4*A*⋯F3^iii^	0.86	2.34	3.047 (3)	139
N4—H4*B*⋯N5^iv^	0.86	2.18	3.038 (3)	178

Symmetry codes: (i) 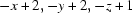; (ii) 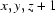; (iii) 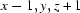; (iv) 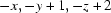.

**Fig. 1.**
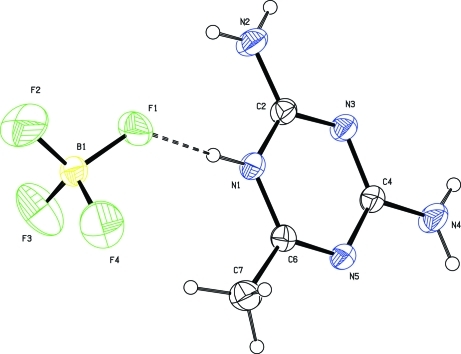
The asymmetric unit of the title compound, showning 30% probability displacement ellipsoids. The dashed line indicate a hydrogen bond.

**Fig. 2.**
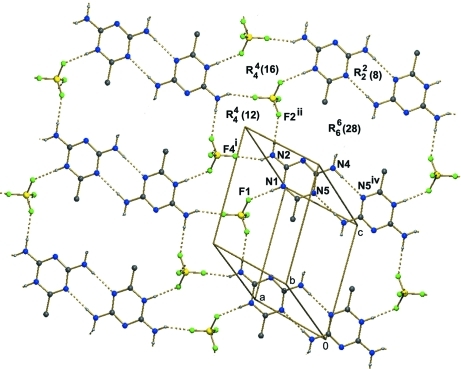
A view of supramolecular layers parallel to the (120) plane formed via N—H···F and N—H···N hydrogen bonds [symmetry codes: (i) x, y, 1+z; (ii) 2-x, 2-y, 1-z; (iv) -x, 1-y, 2-z].

**Fig. 3.**
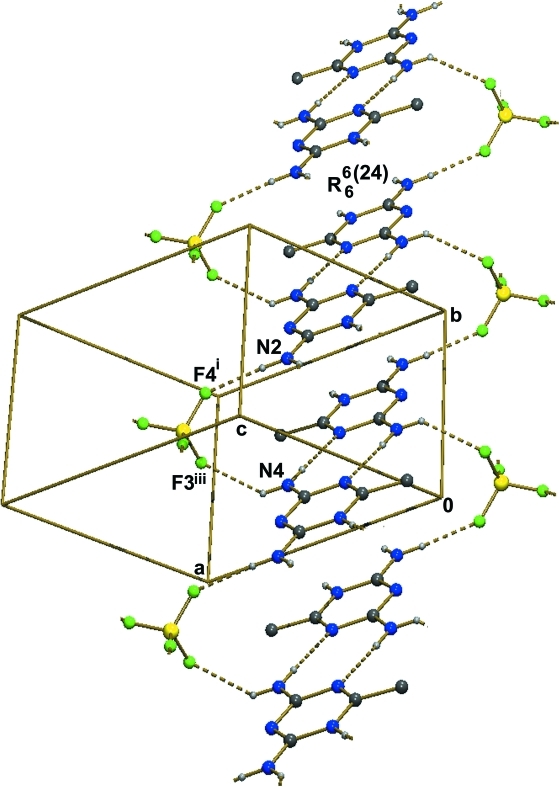
: A view of rings propagating along the b axis formed via N—H···F hydrogen bonds [symmetry codes: (i) x, y, 1+z; (iii) -1+x, y, 1+z].

## supplementary crystallographic information

### Comment

Only a limited number of tetrafluoroborate salts like hydrazinium fluoroborate (Conant *et al.*, 1964), *L*-Histidinium tetrafluoroborate (Gokul Raj *et al.*, 2006), trimethloxosulfonium fluoroborate (Zimmermann *et al.*, 1963) have been reported in the literature. From our laboratory, we have reported the crystal structure of trimethoprim tetrafluoroborate (Hemamalini *et al.*, 2005) and pyrimethamine tetrafluoroborate (Balasubramani *et al.*, 2007), and have analysed their hydrogen bonding patterns. The present investigation concerns the supramolecular patterns exhibited by acetoguanaminium fluoroborate.

The asymmetric unit of the title salt contains one 2,4-diamino-6-methyl-1,3,5-triazin-1-ium (acetoguanaminium) cation and one tetrafluoroborate anion as shown in Fig. 1. The acetoguanaminium cation is protonated at N1. Protonation of the triazine base on the N1 atom is reflected by an increase of the C1—N2—C6 bond angle (119.77 (17)°) with respect to the other C—N—C angles (mean value 115.94 (18)°). The tetrafluoroborate anion shows a slightly distorted tetrahedral geometry (Li *et al.*, 2011). In the asymmetric unit, the acetoguanaminium cation interacts with the tetrafluoroborate anion *via* a nearly linear N—H···F hydrogen bond (Fig. 1, Table 1). Centrosymmetrically-related cations are paired through a pair of N—H···N hydrogen bonds to form a robust *R*_2_^2^(8) ring motif (Etter, 1990; Bernstein *et al.*, 1995) by linking an H atom of the 4-amino group with the N5 atom of the inversion related cation (Table 1). Base pairs are interlinked by *R*_4_^4^(16) ring motifs formed by two of N—H···F hydrogen bonds (Fig. 2; Table 1). The combination of the complementary base pairs (*R*_2_^2^(8)) and *R*_4_^4^(16) motifs generates a supramolecular ribbons parallel to the [211] direction. Adjacent ribbons are interconnected by the alternating occurrence of two different ring motifs such as *R*_4_^4^(12) and *R*_6_^6^(28) forming a supramolecular sheet parallel to the (120) plane as shown in Fig. 2. There is a supramolecular hydrogen bonded ladder generated by the alternating arrangement of *R*_4_^4^(16) and *R*_4_^4^(12) ring motifs which extends along *c* axis. Adjacent sheets are interlinked *via* N—H···F hydrogen bond to form rings with graph set *R*_6_^6^(24) as shown in Fig. 3. These rings propagate along the *b* axis and generates a three dimensional supramolecular network. The structure is further stabilized by nearly face to face π-π stacking interactions between acetoguanaminium rings, with interplanar distance of 3.333 Å, centroid- to-centroid distance of 3.3361 (12)Å and slip angle of 2.46°  (Hunter, 1994). In addition, anion-π contacts are also observed between the acetoguanaminium ring and the F2 and F4 atoms of tetrafluoroborate anion (Cg1···F2^i^ = 3.654 (3) Å; Cg1···F4^i^ = 3.178 (3) Å; Cg1 is the centroid of the N1–N3/C2/C4/C6 ring; symmetry code: (i) 1-x, 2-y, 1-z).

### Experimental

A hot ethanolic solutions of 2,4-diamino-6-methyl-1,3,5-triazine (acetoguanamine; 31 mg; Aldrich) and tetrafluoroboric acid (220 mg of 40% solution; Aldrich) were mixed in a 1:1 molar ratio. The resulting solution was warmed over a water bath for a few minutes and then kept at room temperature for crystallization. After a few days, colourless prismatic crystals suitable for X-ray analysis were obtained.

### Refinement

All hydrogen atoms were positioned geometrically and refined using a riding model, with C—H = 0.96 Å, N—H = 0.86 Å, and with *U*_iso_(H) = 1.2 *U*_eq_(N) or 1.5 *U*_eq_(C).

### Crystal data

**Table:**

C_4_H_8_N_5_^+^·BF_4_^−^	*Z* = 2
*M**_r_* = 212.96	*F*(000) = 216
Triclinic, *P*1	*D*_x_ = 1.603 Mg m^−^^3^
Hall symbol: -P 1	Mo *K*α radiation, λ = 0.71073 Å
*a* = 6.9982 (3) Å	Cell parameters from 2196 reflections
*b* = 8.2887 (4) Å	θ = 2.7–28.4°
*c* = 8.5353 (4) Å	µ = 0.16 mm^−^^1^
α = 63.931 (2)°	*T* = 296 K
β = 83.209 (3)°	Prism, colourless
γ = 85.057 (3)°	0.06 × 0.05 × 0.04 mm
*V* = 441.29 (4) Å^3^	

### Data collection

**Table:**

Bruker SMART APEXII CCD area-detector diffractometer	2196 independent reflections
Radiation source: fine-focus sealed tube	1842 reflections with *I* > 2σ(*I*)
graphite	*R*_int_ = 0.022
φ and ω scans	θ_max_ = 28.4°, θ_min_ = 2.7°
Absorption correction: multi-scan (*SADABS*; Bruker, 2008)	*h* = −9→9
*T*_min_ = 0.990, *T*_max_ = 0.993	*k* = −11→11
8850 measured reflections	*l* = −11→11

### Refinement

**Table:**

Refinement on *F*^2^	Primary atom site location: structure-invariant direct methods
Least-squares matrix: full	Secondary atom site location: difference Fourier map
*R*[*F*^2^ > 2σ(*F*^2^)] = 0.066	Hydrogen site location: inferred from neighbouring sites
*wR*(*F*^2^) = 0.203	H-atom parameters constrained
*S* = 1.09	*w* = 1/[σ^2^(*F*_o_^2^) + (0.1041*P*)^2^ + 0.234*P*] where *P* = (*F*_o_^2^ + 2*F*_c_^2^)/3
2196 reflections	(Δ/σ)_max_ < 0.001
128 parameters	Δρ_max_ = 0.52 e Å^−^^3^
0 restraints	Δρ_min_ = −0.54 e Å^−^^3^

### Special details

**Table:**

Geometry. Bond distances, angles *etc*. have been calculated using the rounded fractional coordinates. All su's are estimated from the variances of the (full) variance-covariance matrix. The cell e.s.d.'s are taken into account in the estimation of distances, angles and torsion angles
Refinement. Refinement on *F*^2^ for ALL reflections except those flagged by the user for potential systematic errors. Weighted *R*-factors *wR* and all goodnesses of fit *S* are based on *F*^2^, conventional *R*-factors *R* are based on *F*, with *F* set to zero for negative *F*^2^. The observed criterion of *F*^2^ > σ(*F*^2^) is used only for calculating -*R*-factor-obs *etc*. and is not relevant to the choice of reflections for refinement. *R*-factors based on *F*^2^ are statistically about twice as large as those based on *F*, and *R*-factors based on ALL data will be even larger.

### Fractional atomic coordinates and isotropic or equivalent isotropic displacement parameters (Å^2^)

**Table:**

	*x*	*y*	*z*	*U*_iso_*/*U*_eq_	
N1	0.5137 (2)	0.7576 (2)	0.7766 (2)	0.0388 (5)	
N2	0.6729 (3)	0.8579 (3)	0.9371 (3)	0.0502 (6)	
N3	0.3894 (2)	0.7141 (2)	1.0587 (2)	0.0408 (5)	
N4	0.1028 (3)	0.5754 (3)	1.1593 (2)	0.0524 (6)	
N5	0.2262 (2)	0.6115 (2)	0.8873 (2)	0.0392 (5)	
C2	0.5241 (3)	0.7772 (3)	0.9261 (3)	0.0376 (5)	
C4	0.2430 (3)	0.6367 (3)	1.0340 (3)	0.0373 (5)	
C6	0.3630 (3)	0.6734 (3)	0.7627 (3)	0.0380 (5)	
C7	0.3631 (4)	0.6521 (4)	0.5987 (3)	0.0569 (8)	
F1	0.7776 (3)	0.9140 (2)	0.4910 (2)	0.0732 (6)	
F2	1.0031 (4)	0.9604 (5)	0.2703 (4)	0.1389 (14)	
F3	0.9060 (4)	0.6845 (3)	0.4382 (3)	0.1076 (9)	
F4	0.7146 (3)	0.8835 (4)	0.2567 (3)	0.1057 (10)	
B1	0.8547 (3)	0.8604 (3)	0.3634 (3)	0.0433 (6)	
H1	0.60230	0.79840	0.69160	0.0470*	
H2A	0.75990	0.89850	0.85050	0.0600*	
H2B	0.68300	0.86990	1.03080	0.0600*	
H4A	0.10620	0.58540	1.25500	0.0630*	
H4B	0.00750	0.52520	1.14560	0.0630*	
H7A	0.28340	0.55470	0.61960	0.0850*	
H7B	0.49230	0.62670	0.56130	0.0850*	
H7C	0.31370	0.76110	0.50920	0.0850*	

### Atomic displacement parameters (Å^2^)

**Table:**

	*U*^11^	*U*^22^	*U*^33^	*U*^12^	*U*^13^	*U*^23^
N1	0.0350 (8)	0.0420 (8)	0.0385 (8)	−0.0107 (6)	0.0081 (6)	−0.0177 (7)
N2	0.0418 (9)	0.0603 (11)	0.0509 (10)	−0.0202 (8)	0.0034 (7)	−0.0250 (9)
N3	0.0361 (8)	0.0496 (9)	0.0389 (8)	−0.0098 (7)	0.0015 (6)	−0.0209 (7)
N4	0.0405 (9)	0.0788 (13)	0.0457 (10)	−0.0223 (9)	0.0106 (7)	−0.0340 (10)
N5	0.0337 (8)	0.0458 (9)	0.0423 (9)	−0.0083 (6)	0.0024 (6)	−0.0232 (7)
C2	0.0340 (9)	0.0364 (9)	0.0413 (10)	−0.0052 (7)	−0.0006 (7)	−0.0158 (7)
C4	0.0325 (9)	0.0412 (9)	0.0383 (9)	−0.0044 (7)	0.0013 (7)	−0.0180 (7)
C6	0.0362 (9)	0.0396 (9)	0.0398 (9)	−0.0040 (7)	0.0017 (7)	−0.0196 (8)
C7	0.0583 (14)	0.0756 (16)	0.0474 (12)	−0.0189 (12)	0.0079 (10)	−0.0366 (12)
F1	0.0826 (11)	0.0843 (11)	0.0659 (10)	−0.0301 (9)	0.0316 (8)	−0.0493 (9)
F2	0.125 (2)	0.192 (3)	0.123 (2)	−0.109 (2)	0.0860 (17)	−0.095 (2)
F3	0.166 (2)	0.0741 (12)	0.0727 (12)	0.0422 (14)	−0.0214 (13)	−0.0284 (10)
F4	0.0973 (16)	0.153 (2)	0.0885 (14)	0.0280 (15)	−0.0463 (12)	−0.0688 (15)
B1	0.0425 (11)	0.0504 (12)	0.0362 (10)	−0.0069 (9)	0.0042 (8)	−0.0191 (9)

### Geometric parameters (Å, °)

**Table:**

F1—B1	1.386 (3)	N5—C4	1.376 (3)
F2—B1	1.331 (4)	N1—H1	0.8600
F3—B1	1.345 (4)	N2—H2A	0.8600
F4—B1	1.361 (3)	N2—H2B	0.8600
N1—C2	1.366 (3)	N4—H4B	0.8600
N1—C6	1.357 (3)	N4—H4A	0.8600
N2—C2	1.319 (3)	C6—C7	1.486 (4)
N3—C4	1.338 (3)	C7—H7B	0.9600
N3—C2	1.325 (3)	C7—H7C	0.9600
N4—C4	1.313 (3)	C7—H7A	0.9600
N5—C6	1.294 (3)		
			
F1···N1	2.758 (2)	C2···F4^i^	3.014 (4)
F1···C6^i^	3.282 (3)	C2···N5^iv^	3.339 (3)
F2···N2^ii^	2.800 (4)	C2···C4^iv^	3.561 (4)
F3···N4^iii^	3.047 (3)	C2···C6^iv^	3.591 (4)
F3···N4^iv^	3.149 (3)	C4···N1^iv^	3.353 (3)
F3···F3^v^	3.000 (4)	C4···C2^iv^	3.561 (4)
F4···C2^i^	3.014 (4)	C6···F1^i^	3.282 (3)
F4···N2^vi^	2.877 (4)	C6···N3^iv^	3.317 (3)
F4···N1^i^	3.167 (4)	C6···C2^iv^	3.591 (4)
F1···H7C^i^	2.7100	C6···H4B^x^	3.0300
F1···H1	1.9000	B1···H1	2.9900
F2···H2A^ii^	2.0100	H1···H2A	2.2900
F3···H4A^iv^	2.5900	H1···H7B	2.3700
F3···H7A^vii^	2.7200	H1···B1	2.9900
F3···H4A^iii^	2.3400	H1···F1	1.9000
F4···H2B^vi^	2.0200	H2A···F2^ii^	2.0100
N1···F4^i^	3.167 (4)	H2A···H1	2.2900
N1···C4^iv^	3.353 (3)	H2B···F4^viii^	2.0200
N1···F1	2.758 (2)	H4A···F3^iv^	2.5900
N2···F4^viii^	2.877 (4)	H4A···F3^ix^	2.3400
N2···F2^ii^	2.800 (4)	H4B···H7A^x^	2.5900
N3···C6^iv^	3.317 (3)	H4B···N5^x^	2.1800
N4···F3^ix^	3.047 (3)	H4B···C6^x^	3.0300
N4···F3^iv^	3.149 (3)	H7A···F3^vii^	2.7200
N4···N5^x^	3.038 (3)	H7A···H4B^x^	2.5900
N5···N4^x^	3.038 (3)	H7B···H1	2.3700
N5···C2^iv^	3.339 (3)	H7C···F1^i^	2.7100
N5···H4B^x^	2.1800		
			
C2—N1—C6	119.77 (17)	N3—C4—N4	118.8 (2)
C2—N3—C4	116.00 (18)	N1—C6—C7	117.1 (2)
C4—N5—C6	115.89 (18)	N5—C6—C7	121.1 (2)
C6—N1—H1	120.00	N1—C6—N5	121.9 (2)
C2—N1—H1	120.00	C6—C7—H7A	109.00
C2—N2—H2A	120.00	C6—C7—H7B	109.00
H2A—N2—H2B	120.00	C6—C7—H7C	109.00
C2—N2—H2B	120.00	H7A—C7—H7B	109.00
H4A—N4—H4B	120.00	H7A—C7—H7C	109.00
C4—N4—H4B	120.00	H7B—C7—H7C	109.00
C4—N4—H4A	120.00	F1—B1—F2	110.0 (3)
N1—C2—N2	118.6 (2)	F1—B1—F3	109.8 (2)
N2—C2—N3	120.5 (2)	F1—B1—F4	107.9 (2)
N1—C2—N3	120.9 (2)	F2—B1—F3	111.7 (3)
N3—C4—N5	125.55 (19)	F2—B1—F4	109.6 (2)
N4—C4—N5	115.7 (2)	F3—B1—F4	107.8 (3)
			
C6—N1—C2—N2	179.3 (2)	C2—N3—C4—N4	−178.4 (2)
C6—N1—C2—N3	0.4 (3)	C2—N3—C4—N5	2.8 (3)
C2—N1—C6—N5	0.6 (3)	C6—N5—C4—N3	−1.9 (3)
C2—N1—C6—C7	−178.4 (2)	C6—N5—C4—N4	179.3 (2)
C4—N3—C2—N1	−2.0 (3)	C4—N5—C6—N1	0.1 (3)
C4—N3—C2—N2	179.1 (2)	C4—N5—C6—C7	179.0 (2)

Symmetry codes: (i) −*x*+1, −*y*+2, −*z*+1; (ii) −*x*+2, −*y*+2, −*z*+1; (iii) *x*+1, *y*, *z*−1; (iv) −*x*+1, −*y*+1, −*z*+2; (v) −*x*+2, −*y*+1, −*z*+1; (vi) *x*, *y*, *z*−1; (vii) −*x*+1, −*y*+1, −*z*+1; (viii) *x*, *y*, *z*+1; (ix) *x*−1, *y*, *z*+1; (x) −*x*, −*y*+1, −*z*+2.

### Hydrogen-bond geometry (Å, °)

**Table:**

*D*—H···*A*	*D*—H	H···*A*	*D*···*A*	*D*—H···*A*
N1—H1···F1	0.86	1.90	2.758 (2)	173
N2—H2A···F2^ii^	0.86	2.01	2.800 (4)	152
N2—H2B···F4^viii^	0.86	2.02	2.877 (4)	177
N4—H4A···F3^ix^	0.86	2.34	3.047 (3)	139
N4—H4B···N5^x^	0.86	2.18	3.038 (3)	178

Symmetry codes: (ii) −*x*+2, −*y*+2, −*z*+1; (viii) *x*, *y*, *z*+1; (ix) *x*−1, *y*, *z*+1; (x) −*x*, −*y*+1, −*z*+2.
